# Cocaine remodels m^6^A RNA-dependent signaling to drive locomotor plasticity in *Drosophila melanogaster*

**DOI:** 10.3389/fncel.2026.1810118

**Published:** 2026-04-15

**Authors:** Ana Filošević Vujnović, Nina Milotić, Bobana Samardžija, Marko Rubinić, Rozi Andretić Waldowski, Alessia Soldano

**Affiliations:** 1 Faculty of Biotechnology and Drug Development, University of Rijeka, Rijeka, Croatia; 2 Neuroscience Area, Scuola Internazionale Superiore di Studi Avanzati (SISSA), Trieste, Italy

**Keywords:** cocaine, *Drosophila melanogaster*, locomotor sensitization, m6A RNA modification, MeRIP-seq, non-coding RNA

## Abstract

N^6^-methyladenosine (m^6^A) is a dynamic RNA modification that regulates RNA stability, processing, and translation and is increasingly recognized as a key modulator of neuronal plasticity. However, how psychostimulant exposure reshapes m^6^A-dependent regulatory networks across coding and non-coding RNA species remains poorly understood. We investigated the impact of volatilized cocaine (vCOC) exposure on m^6^A RNA methylation, m^6^A pathway components, transcriptome, and cocaine-induced locomotor sensitization in *Drosophila melanogaster*. Acute vCOC administration significantly increased global m^6^A levels in total and poly(A)-enriched RNA, with a stronger effect in polyadenylated transcripts. This increase occurred without changes in the m^6^A methyltransferases Mettl3 and Mettl14 transcripts, but was accompanied by robust upregulation of the levels of m^6^A reader YTHDC and YTHDF transcripts. Genetic and cell-type-specific analyses revealed distinct and context-dependent roles for m^6^A writers and readers in neurons and glia, with m^6^A readers being essential for vCOC-induced locomotor sensitization. Integration of RNA-seq and MeRIP-seq demonstrated that vCOC selectively amplifies m^6^A modification of regulatory and plasticity-associated RNA classes, including mRNAs involved in RNA processing, antisense RNAs, long non-coding RNAs, and transposable element-derived transcripts. In contrast, m^6^A-modified RNAs shared in CTRL and vCOC were enriched for core metabolic and mitochondrial pathways, such as oxidative phosphorylation. Notably, vCOC increased m^6^A modification of non-coding RNAs and transposable elements with minimal overlap with control conditions, indicating cocaine-induced engagement of epitranscriptomic regulation at multiple layers of the transcriptome. Together, these findings reveal that cocaine exposure reinforces an m^6^A-defined regulatory RNA network, spanning coding and non-coding transcripts that is mechanistically linked to m^6^A reader-dependent behavioral plasticity.

## 1 Introduction

Cocaine is a potent psychostimulant that induces robust locomotor activation and behavioral sensitization across species, reflecting conserved effects on neural circuits controlling arousal and movement (dos Santos-Baldaia et al., 2023; Runegaard et al., 2018; Steketee and Kalivas, 2011; Robinson and Berridge, 2008; Wolf, 1998). Locomotor sensitization (LS) is induced by repeated exposure to the same dose of the drug, which leads to progressively increased movement or activity. In *Drosophila melanogaster*, volatilized cocaine (vCOC) reliably evokes LS, which can be precisely quantified using the FlyBong assay, a system that allows controlled drug delivery and high-throughput behavioral analysis, used in dissecting molecular mechanisms underlying cocaine-induced behavioral plasticity (Filošević Vujnović et al., 2023b; Filošević et al., 2018; McClung and Hirsh, 1998).

LS, as an enduring behavioral adaptation, indicates persistent molecular remodeling of neural circuits engaged by repeated cocaine exposure. Although transcriptional and epigenetic mechanisms mediating cocaine responses have been extensively studied in mammal models (Wang et al., 2025; Filošević Vujnović et al., 2024; Hamilton and Nestler, 2019; De Sa Nogueira et al., 2019; Maze and Nestler, 2011), the role of post-transcriptional RNA regulation in shaping cocaine-induced LS remains largely unexplored. Post-transcriptional mechanisms are uniquely positioned to confer rapid, reversible, and cell-type-specific control over gene expression, required for long-lasting behavioral plasticity.

N^6^-methyladenosine (m^6^A) is the most abundant internal modification of eukaryotic RNA, dynamically regulating RNA stability, translation, localization, and decay (He and He, 2021; Meyer and Jaffrey, 2014). m^6^A deposition is catalyzed by the methyltransferase complex Mettl3/Mettl14, while downstream effects are mediated by “reader” proteins, including nuclear YTHDC and cytoplasmic YTHDF families, which interpret m^6^A to control RNA fate (Worpenberg et al., 2021; Kan et al., 2021; Fu and Zhuang, 2020; Liu et al., 2014). Despite growing evidence that m^6^A-mediated RNA regulation plays a critical role in neuronal development, synaptic plasticity, and experience-dependent gene expression, its contribution to drug-induced behavioral adaptations remains poorly defined (Hronova et al., 2025; Sokpor et al., 2021; Liu and Si, 2024; Widagdo et al., 2016). While cocaine exposure is known to elicit widespread transcriptional and epigenetic reprogramming in reward-related circuits, whether cocaine alters m^6^A deposition or reader-mediated modulation of RNA fate to drive LS is unknown (Xue et al., 2021; Bu et al., 2012; Zhou et al., 2011; Hollander et al., 2010). Moreover, the identity of m^6^A-modified transcripts and reader proteins that link repeated cocaine exposure to persistent behavioral change has not been investigated. This gap limits our understanding of how post-transcriptional RNA mechanisms interface with established transcriptional mechanisms that stabilize cocaine-induced neural plasticity.

Beyond protein-coding transcripts, accumulating evidence indicates that non-coding RNA species are critical regulators of drug-induced plasticity (Li et al., 2022; Seyednejad and Sartor, 2022; Jung, 2020; Sartor et al., 2012). Long non-coding RNAs (lncRNAs) are increasingly recognized as modulators of chromatin state, transcription, and synaptic function, and several lncRNAs have been reported to be altered by cocaine exposure in mammalian brain regions, such as the nucleus accumbens and prefrontal cortex (Xu et al., 2020; Bannon et al., 2015). In parallel, transposable elements (TEs), which constitute a substantial fraction of eukaryotic genomes, are emerging as dynamic and activity-responsive components of neuronal transcriptomes (Barter and Cho, 2025; He and Lan, 2021). Psychostimulant exposure, including cocaine, has been shown to influence TE expression and mobilization in the brain, potentially contributing to long-lasting changes in gene regulation, neuronal diversity, and circuit identity (Doyle et al., 2017; Maze et al., 2011). However, whether lncRNAs and TE-derived RNAs participate in cocaine-induced behavioral plasticity in invertebrate models, and how their regulation is coordinated at the post-transcriptional level, remains unknown.

m^6^A provides a unifying mechanism linking protein-coding genes, lncRNAs, and TE-derived RNAs into a single regulatory framework for cocaine-induced plasticity. In neurons, m^6^A is essential for synaptic plasticity, activity-dependent gene expression, and learning and memory (Yu et al., 2018; Widagdo et al., 2016; Shi et al., 2018), while glial m6A signalling seems to regulate glial cell development, metabolic support, neurotransmitter clearance, and neuron-glia communication (You et al., 2022; Wang et al., 2021; Wang et al., 2025). Importantly, m^6^A has been shown to regulate the stability, processing, and function of lncRNAs and to modulate transcripts derived from TE, suggesting that epitranscriptomic mechanisms may coordinate multiple RNA classes within cocaine-responsive neural circuits (He and Lan, 2021; Jung, 2020; Xu et al., 2020; Maze et al., 2011). Despite these established roles, whether cocaine engages m^6^A-dependent regulation of lncRNAs and TE-derived RNAs to drive persistent behavioral adaptations such as LS remains largely unexplored.

Here, we investigated how m^6^A RNA methylation modulates cocaine-induced LS in *Drosophila melanogaster*. We hypothesized that vCOC alters m^6^A signaling to drive LS, and that both neuronal and glial m^6^A pathways contribute to this process. To test this hypothesis, we combined biochemical, molecular, genetic, and transcriptomic approaches. We first quantified global m^6^A levels and assessed mRNA expression of core m^6^A writers and readers following vCOC exposure. We then determined the behavioral consequences of disrupting m^6^A signaling using genetic mutants and cell-type-specific knockdowns in neurons and glia. Finally, using polyA-enriched RNA samples, we performed RNA-seq and m^6^A RNA immunoprecipitation sequencing (MeRIP-seq) to examine how cocaine remodels m^6^A-marked transcriptomes and associated pathways, with particular attention to differential regulation of lncRNAs and TE-derived transcripts.

## 2 Materials and methods

### 2.1 Fly line and breeding

We used 3–5 days old male flies of the following genotypes: wild type *CantonS* (Bloomington code: BL64349), mutant lines Mettl3 *null*, YTHDF^ΔYTH^, YTHDC^def^ and YTHDC^Δ^ (Worpenberg et al., 2021), and RNAi-mediated knockdown lines *UAS-Mettl3* RNAi (BL80450), *UAS-YTHDF* RNAi (BL55151), *UAS-YTHDC* RNAi (BL34627) and *UAS-LUC* RNAi (BL31603). Knockdowns were driven by Elav-Gal4 (BL458) and Repo-Gal4: UAS-CD8-GFP (Read et al., 2009) to target m^6^A related genes in neurons and glial cells, respectively. Flies were reared at 25 °C, 70% humidity, and a 12-h light: 12-h dark cycle on standard cornmeal-agar medium containing inactivated dry yeast, sugar, nipagin, and propionic acid.

### 2.2 Sample preparation for quantitative analysis of m^6^A methylation

**Figure 1:**
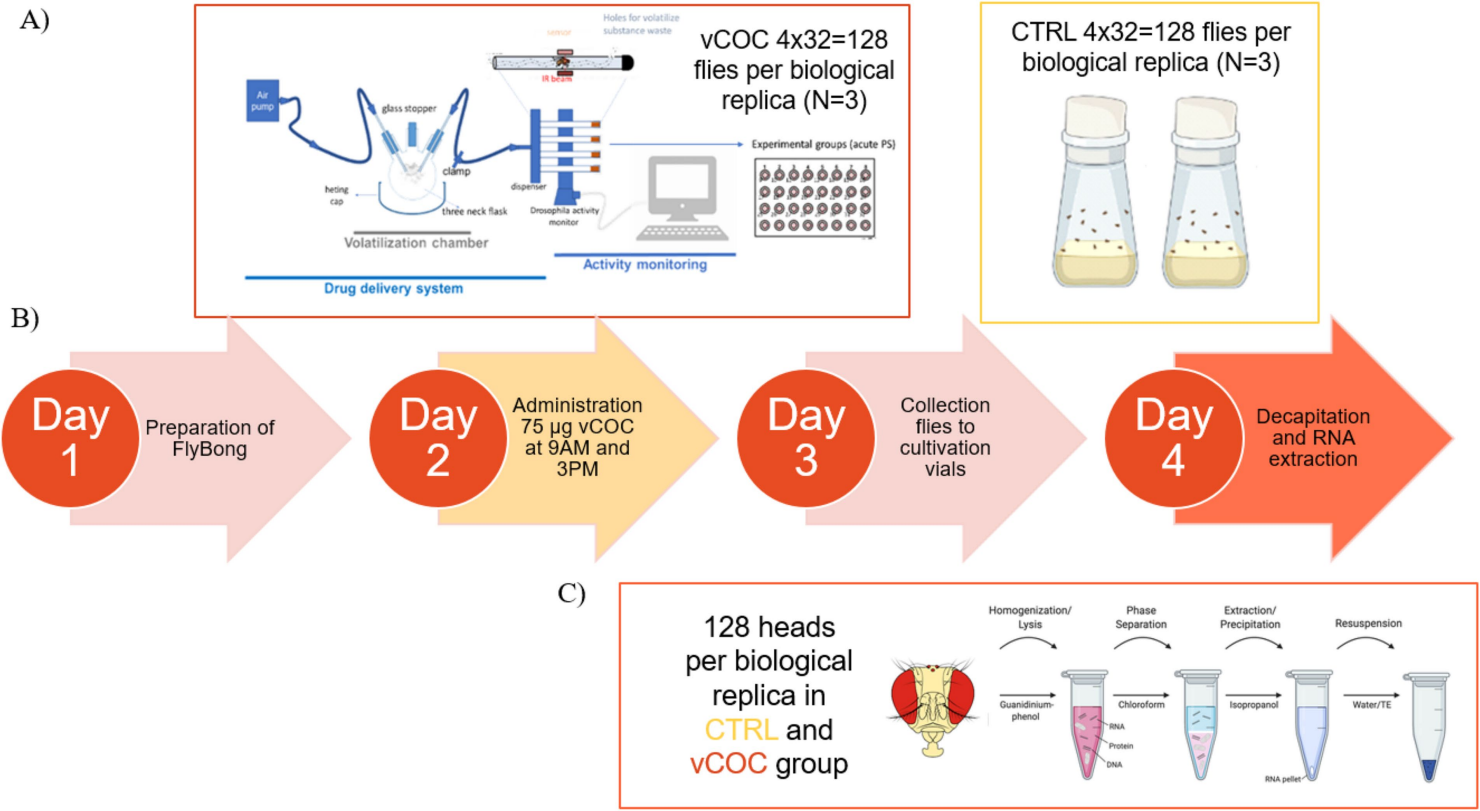
Sample preparation for RNA analysis. **(A)** Fly Bong assay and experimental groups. **(B)** Timeline of experimental procedure. **(C)** RNA extraction for downstream analysis. Created with BioRender.com and adapted from Filošević et al., 2018.

Flies were divided into two groups control (CTRL) and experimental (vCOC). The CTRL group consisted of 3–5-day-old male *wild type (wt)* flies of *CantonS* background collected from cultivation bottles, while the vCOC group consisted of 3–5-day-old male *wt* exposed to two doses of volatilized cocaine (75 μg, 6 h apart) using the FlyBong system (Figure 1A). CTRL flies and 48 h after first vCOC exposure flies were decapitated using forceps and CO_2_ anesthesia at 9 a.m. Heads were collected with a brush into ice-cold PBSX1 (Figure 1B). Samples were centrifuged for 3 min at 4 °C and 2,500 rpm to allow the heads to settle at the bottom of a 1.5 mL Eppendorf tube. The supernatant was removed, and the samples were either processed immediately for total RNA extraction or stored at −80 °C.

### 2.3 Total RNA extraction and quantification

Total RNA was extracted from fly heads using TRIzol reagent (Invitrogen). Samples were homogenized in 200 μL TRIzol, followed by the addition of 300 μL TRIzol and incubation for 5 min at room temperature. Phase separation was achieved with 100 μL chloroform and centrifugation at 12,000 rpm for 15 min at 4 °C. The aqueous phase (~200 μL) was mixed with 250 μL isopropanol and incubated overnight at −80 °C (Figure 1C). RNA was pelleted by centrifugation (12,000 rpm, 10 min, 4 °C), washed twice with 70% ethanol, air-dried, and resuspended in 15 μL RNase-free water preheated to 60 °C. RNA concentration and purity were determined using a NanoDrop spectrophotometer (DeNovix DS-11) at 260 nm. The 260/280 and 260/230 absorbance ratios were used to assess RNA quality, while RNA samples were stored at −80 °C until further use.

### 2.4 Poly(A) RNA enrichment

Poly(A) + RNA was enriched from total RNA using the NEBNext Poly(A) mRNA Magnetic Isolation Module (NEB #E7490) according to the manufacturer’s instructions. This step was performed to deplete rRNA, tRNA and other RNA that contain m^6^A, and to focus subsequent MeRIP-seq analyses on mRNA and lncRNA. Briefly, 5 μg of total RNA was incubated with 20 μL of oligo(dT)25 magnetic beads pre-washed with RNA Binding Buffer (2×). RNA binding was performed by mixing 50 μL of diluted RNA with 50 μL RNA Binding Buffer, followed by incubation at 80 °C for 2 min and 25 °C for 5 min. Bound RNA was eluted in 17 μL of Tris buffer at 80 °C for 2 min and cooled to 25 °C. Approximately 15 μL of poly(A) + RNA was recovered and stored at −80 °C. RNA quantity and quality were verified using a Bioanalyzer (Pico chip) or fluorescent assay (DeNovix KIT-RNA-1-NS).

### 2.5 m^6^A RNA colorimetric quantification

Global m^6^A levels were quantified using the EpiQuik™ m^6^A RNA Colorimetric Methylation Quantification Kit (Epigentek) according to the manufacturer’s protocol. The assay was performed using 100–300 ng of total RNA or 100 ng of poly(A) + RNA. RNA samples were bound to strip wells at 37 °C for 90 min, washed, and sequentially incubated with capture and detection antibodies, enhancer, and developer solutions. Absorbance was measured at 450 nm using a Thermo Scientific Multiskan FC microplate reader. A standard curve (0.02–1 ng m^6^A) was generated using the positive control to calculate absolute m^6^A content. Results were expressed as the percentage of m^6^A RNA (mean ± SEM). Statistical significance was assessed using a two-tailed unpaired *t*-test in GraphPad Prism.

### 2.6 cDNA synthesis and quantitative real-time PCR (RT-qPCR)

cDNA was synthesized from 0.5 to 2 μg of total RNA with the same amount within the same experiment, using the High-Capacity cDNA Reverse Transcription Kit (Thermo Fisher Scientific) following the manufacturer’s instructions. The 20 μL reaction contained 10 μL 2 × RT master mix and 10 μL RNA template. Reverse transcription was performed at 25 °C for 10 min, 37 °C for 120 min, and 85 °C for 5 min, followed by storage at −25 °C. RT-qPCR was carried out using SsoAdvanced Universal SYBR Green Supermix (Bio-Rad) on a CFX96 Touch Real-Time PCR Detection System (Bio-Rad). Each 10 μL reaction contained 5 μL SYBR mix, 0.5 μL of each primer (10 μM), 1 μL cDNA (7 ng), and 3 μL nuclease-free water. Cycling conditions were 95 °C for 3 min, followed by 40 cycles of 95 °C for 15 s and 60 °C for 30 s. Primer specificity was verified by BLAST and melt-curve analysis (Table 1). Relative gene expression was calculated using the 2^^–ΔΔCt^ method with Rp49-1 as the reference gene.

**Table 1:** Primers (Merck) used in experiments with vCOC and control flies.

Gene	Forward (5′- > 3′)	Reverse (5′- > 3′)	Reference
Mettl3	AAGGAACTCGTTGAGGCTGA	CACCTGTGTGGAGACAATGG	Lence et al. (2016)
Mettl14	AAGCGTCGTTTGCTTTTAGC	GCATTACCCAAAGCCTTTTTC	Lence et al. (2016)
YTHDF	CCGAGAAAGTGCACAAGGAT	AAACCTTGGCTCTGCTGAAG	Lence et al. (2016)
YTHDC	GGTCGTGATTTGATCCTCTG	TCGAACTCACTCCCATACTC	Zhang et al. (2025)
RP49-1	ATGCTAAGCTGTCGCACAAA	CGATGTTGGGCATCAGATACT	Ruaud et al. (2011)

### 2.7 Motor-activating effects using FlyBong assay

Locomotor responses to volatilized cocaine (vCOC) were assessed using the FlyBong assay as previously described (Filošević et al., 2018). The setup comprised a vertical TriKinetics *Drosophila* Activity Monitoring (DAM) system coupled to a drug-delivery apparatus consisting of a 250 mL three-neck flask and heat cap (LabHEAT KM-G) serving as the volatilization chamber (Figure 1A). A 10 mg/mL cocaine hydrochloride solution (≥97.5%, Sigma-Aldrich) was prepared in 96% ethanol (VWR), and 75 μL was added to the flask 4–6 h before exposure to allow ethanol evaporation. The flask was heated for 8 min at 185 °C to volatilize cocaine, and the resulting vapor was delivered via an air pump to 32 polycarbonate tubes containing single flies and food. Each fly received two 1-min vCOC exposures (9 a.m. and 3 p.m.). Locomotor activity was recorded as infrared beam crossings per minute using DAMS, averaged over 30 min before and after each exposure. A control group was exposed to a hot air puff within the FlyBong system. Locomotor response was defined as the average locomotor activity during the 30 min following the first exposure at 9 a.m. minus the average 30-min baseline activity before exposure, representing locomotor 1st response after the first exposure. Similarly, 2nd locomotor response was calculated as the average locomotor activity during the 30 min following the second exposure at 3 p.m. minus the 30-min baseline before exposure. Locomotor sensitization (LS) was assessed by comparing responses from the first, 1st, and second, 2nd, exposures, reflecting the stepwise increase in locomotor activity across exposures. For each genotype we used 3–5 days old male flies and the experiment was repeated twice with 16 flies.

### 2.8 MeRIPseq pipeline

#### 2.8.1 RNA fragmentation and spiking

**Figure 2:**
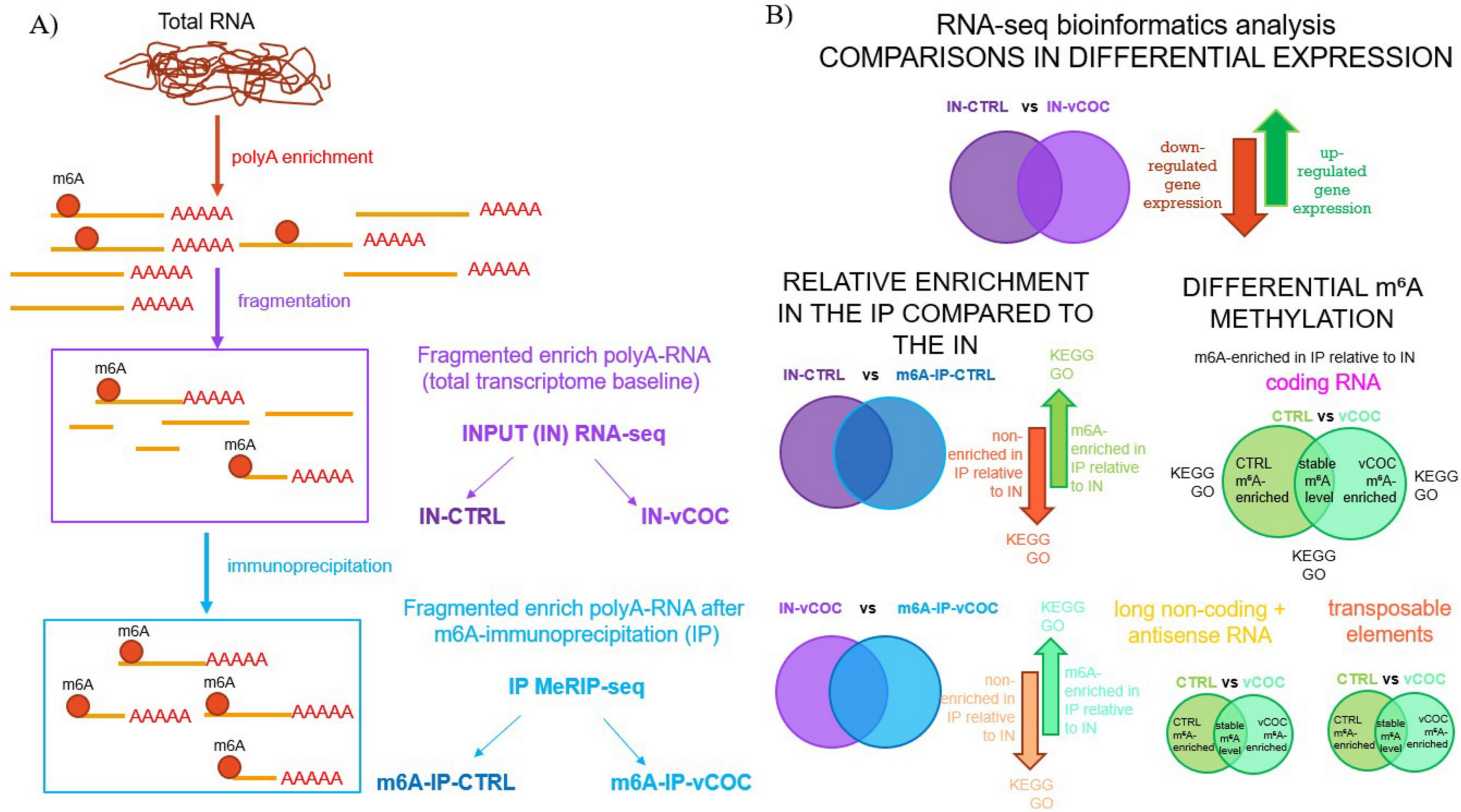
MeRIP-seq pipeline for sample preparation **(A)** and bioinformatic data analysis **(B)**.

Poly(A) + RNA (≈100 ng) was fragmented using the NEBNext Magnesium RNA Fragmentation Module (NEB #E6150) by incubation at 94 °C for 5 min, followed by addition of Stop Solution (10×) and cooling on ice (Figure 2A). To monitor immunoprecipitation (IP) efficiency, each sample prior to IP was spiked in with 1 μL of synthetic m^6^A-modified Gaussian luciferase RNA (+m^6^A RNA) and 1 μL of unmodified Cypridina luciferase RNA (−m^6^A RNA) (1:1000 dilution). Before moving to the immunoprecipitation, aliquots of fragmented Poly(A) RNA were taken for quantity and quality evaluation with the Bioanalyzer, input (IN), and qPCR controls. Fragmented RNA was purified either by ethanol precipitation or using the Monarch RNA Clean-up Kit (NEB #T2030). For ethanol precipitation, RNA was mixed with 3 M sodium acetate (pH 5.2) and ethanol, incubated overnight at −80 °C, centrifuged at 14,000 rpm for 25 min at 4 °C, washed twice with 70% ethanol, and resuspended in 13.5 μL RNase-free water.

#### 2.8.2 m^6^A RNA immunoprecipitation (MeRIP)

Immunoprecipitation was performed using the EpiMark N^6^-Methyladenosine Enrichment Kit (NEB #E1610S). Protein G magnetic beads were washed and incubated with anti-m^6^A antibody (rabbit monoclonal) for 2 h at 4 °C. Antibody-coated beads were then incubated with fragmented, spiked RNA for 2 h at 4 °C with rotation. Beads were washed with reaction buffer and m^6^A-enriched RNA was eluted using the Monarch RNA Clean-up system following the manufacturer’s protocol. Purified RNA was stored at −80 °C until further use (Figure 1C). To assess IP efficiency, 1 μL of input and IP fractions was reverse transcribed and analyzed by RT-qPCR using primers for GLuc (+m^6^A) and CLuc (−m^6^A) control RNAs. Comparable Ct values between input and IP were observed for GLuc, while CLuc showed a ~ 3 Ct increase in IP samples, confirming specific m^6^A enrichment. RNA integrity and concentration before and after IP were evaluated using the Agilent 2100 Bioanalyzer with the RNA 6000 Pico Kit (Agilent Technologies). RIN values of 2.5–3.5 were consistent with expected fragmentation, and RNA concentrations were sufficient for subsequent RNA-seq library preparation.

#### 2.8.3 Library preparation and sequencing

RNA-seq libraries were prepared by the company IGATech (Udine, Italy) using the Ovation SoLo RNA-seq Library Preparation Kit (Tecan Genomics, Redwood City, CA) according to the manufacturer’s protocol (library type: fr-second strand). RNA quantity and integrity were assessed using either the Agilent 2100 Bioanalyzer RNA assay or the TapeStation RNA assay (Agilent Technologies, Santa Clara, CA). Final library quality and concentration were verified with a Qubit 3.0 Fluorometer (Invitrogen, Carlsbad, CA) and the Agilent Bioanalyzer DNA assay. Prepared libraries were subsequently processed for sequencing and run in paired-end 150 bp mode on NovaSeq X Plus platform (Illumina, San Diego, CA).

#### 2.8.4 RNA-Seq bioinformatics analysis

Standard RNA-seq bioinformatics processing was performed by the company IGATech (Udine, Italy) following established base calling and demultiplexing carried out using Illumina BCL Convert v3.9.31. Adapter sequences and low-quality bases were removed with Cutadapt v1.11, and read deduplication was performed with UMI-tools using 8-base unique molecular identifiers (UMIs) to distinguish true PCR duplicates from independently generated library molecules. Cleaned reads were aligned to the *Drosophila melanogaster* reference genome (BDGP6.54, release 115) using STAR3 with default parameters, allowing accurate detection of splice junctions. Transcript assembly and quantification were conducted with StringTie4 under default settings to generate gene- and transcript-level expression estimates. Quality control metrics, including strand specificity, gene body coverage, read distribution, and insert-size statistics for paired-end data, were assessed using the RSeQC5 package. Differential expression and differential m^6^A enrichment analyses of RNA-seq and MeRIP-seq data were performed using DESeq2 (Anders and Huber, 2010). Statistical significance was defined using an adjusted *p*-value (padj) < 0.05 and an absolute log₂ fold change (|log₂FC|) > 1. Gene Ontology (GO) enrichment analysis was performed using ShinyGO v0.85.1 (Ge et al., 2020). Lists of significantly upregulated (padj < 0.05, log₂FC > 0) and downregulated (padj < 0.05, log₂FC < 0) genes from each comparison were analyzed separately. *Drosophila melanogaster* was specified as the reference species (taxonomy ID: 7227) using ENSEMBL BDGP6.46 annotation. The background gene set comprised all genes detected in the respective dataset. Analyses were conducted with an FDR cutoff of 0.10, a maximum of 20 pathways displayed, and redundancy reduction enabled. Overlap analysis between datasets was performed using Venny 2.1.^1^ Data visualization and statistical plotting were conducted in R (v4.3.2) using the ggplot2, dplyr, and tidyverse packages. Differential gene expression analysis was performed using input (IN) fragmented poly(A) + RNA, representing the total transcriptome baseline, from control (CTRL) and vCOC-treated groups. Results were visualized as volcano plots, and genes showing significant changes between groups were classified as up-regulated or down-regulated in response to vCOC treatment (Figure 2B). To assess m^6^A RNA methylation, fragmented poly(A) + RNA immunoprecipitated with an anti-m^6^A antibody (IP) was compared to the corresponding input (IN) samples. This analysis was conducted separately for CTRL and vCOC conditions to identify m^6^A-enriched RNA regions (IP-enriched relative to IN), representing putative m^6^A-modified transcripts (Figure 2B). RNA regions that did not show significant enrichment in IP relative to IN were classified as non-enriched and considered background signal. Differential m^6^A methylation between CTRL and vCOC groups was evaluated by comparing IP/IN enrichment ratios across conditions. RNA regions showing increased or decreased enrichment were classified as hypermethylated or hypomethylated, respectively (Figure 2B). Pathway enrichment was calculated separately for differentially expressed genes, m^6^A-enriched RNA regions and hyper/hypomethylated RNA.

### 2.9 Statistical data analysis

All statistical analyses and data visualizations were conducted using GraphPad Prism (version 10.6.1, La Jolla, CA, USA). Normality of the data was assessed with Bartlett’s test or Brown-Forsythe’s test as appropriate. Group comparisons were performed using either unpaired *t*-test or one-way ANOVA followed by Tukey’s *post hoc* multiple comparisons test, depending on the data set. Differences were considered statistically significant at *p* < 0.05.

## 3 Results

### 3.1 Volatilized cocaine increases m^6^A RNA

**Figure 3:**
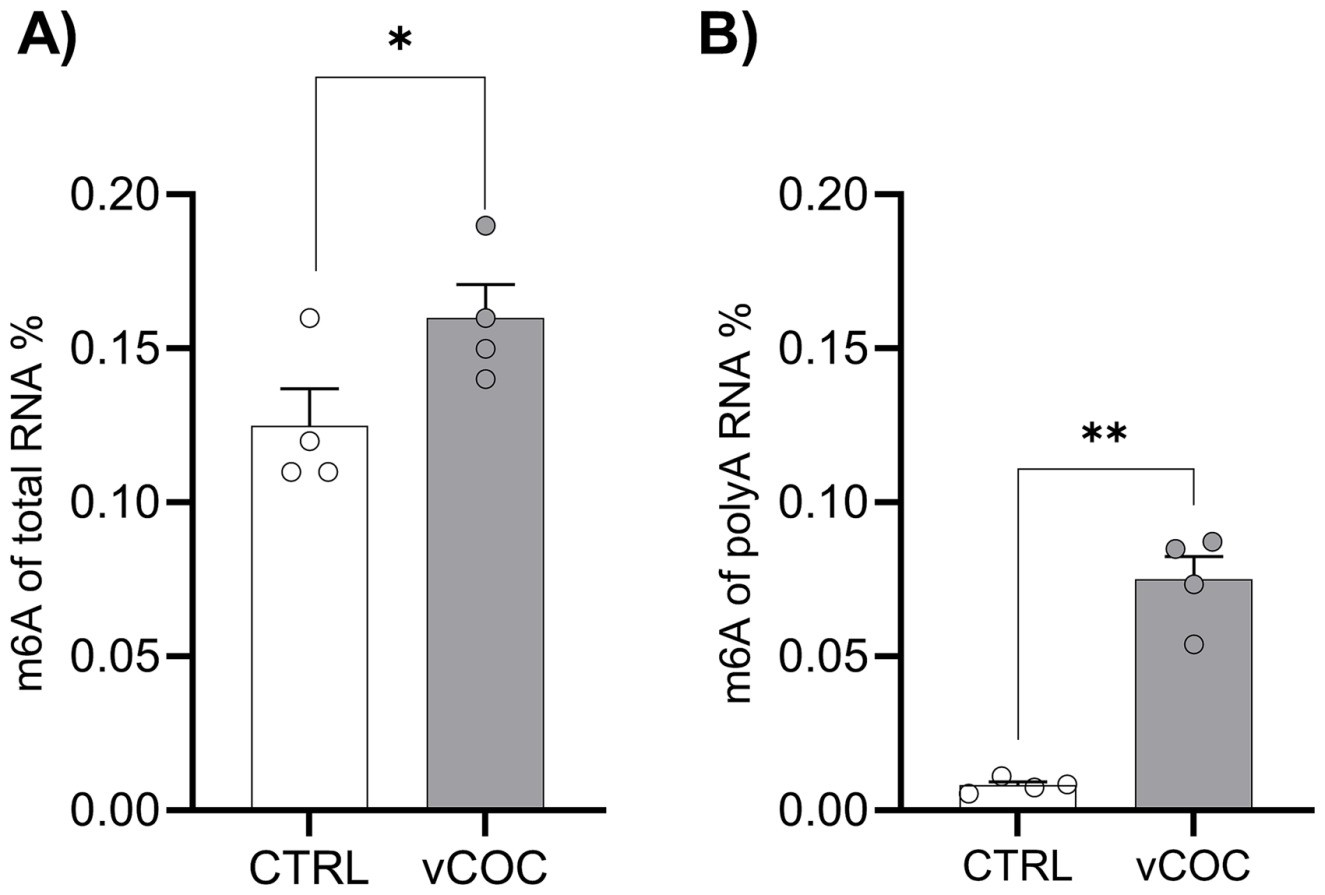
Volatilized cocaine exposure increased m^6^A levels in total RNA and polyA-enriched RNA samples. Colorimetric quantification of m^6^A RNA methylation was performed on four biological replicates with two technical duplicates for each condition: control (CTRL) and experimental (vCOC) for **(A)** total RNA (200 ng), and **(B)** polyA-enriched RNA (100 ng). The CTRL group consisted of 3–5-day-old male WT flies collected from cultivation bottles, while the vCOC group consisted of 3–5-day-old male WT flies exposed to two doses of volatilized cocaine (75 μg, 6 h apart) using the FlyBong system. Data are presented as mean ± SEM of m^6^A RNA percentage. Statistical analysis was performed using a two-tailed unpaired *t*-test with calculations carried out in GraphPad Prism software. * < 0.5.

To determine whether volatilized cocaine (vCOC) administration influences m^6^A RNA levels, 3–5-day-old male wild type (*wt*) flies were exposed to two 75 μg doses of vCOC using the FlyBong system. This vCOC paradigm enables precise and reproducible drug delivery to large groups of flies at the same time. Flies were decapitated 48 h after two vCOC exposures, and total head RNA was isolated. m^6^A levels were measured in both total RNA and polyA-enriched RNA using a colorimetric assay and quantified as proportion of m^6^A RNA in total RNA, or in polyA-enriched RNA. vCOC exposure led to a significant increase in m^6^A levels in both total (Figure 3A) and polyA-enriched RNA samples (Figure 3B). These results suggest that vCOC enhanced m^6^A methylation. The vCOC effect was more pronounced in polyA-enriched RNA samples compared to the more diverse total RNA. This selective increase in m^6^A levels may contribute to post-transcriptional regulation of specific mRNAs or lncRNAs in response to vCOC exposure, potentially affecting RNA stability, translation, or localization contained within polyA-enriched RNA samples.

### 3.2 Volatilized cocaine increases expression of m^6^A reader genes

**Figure 4:**
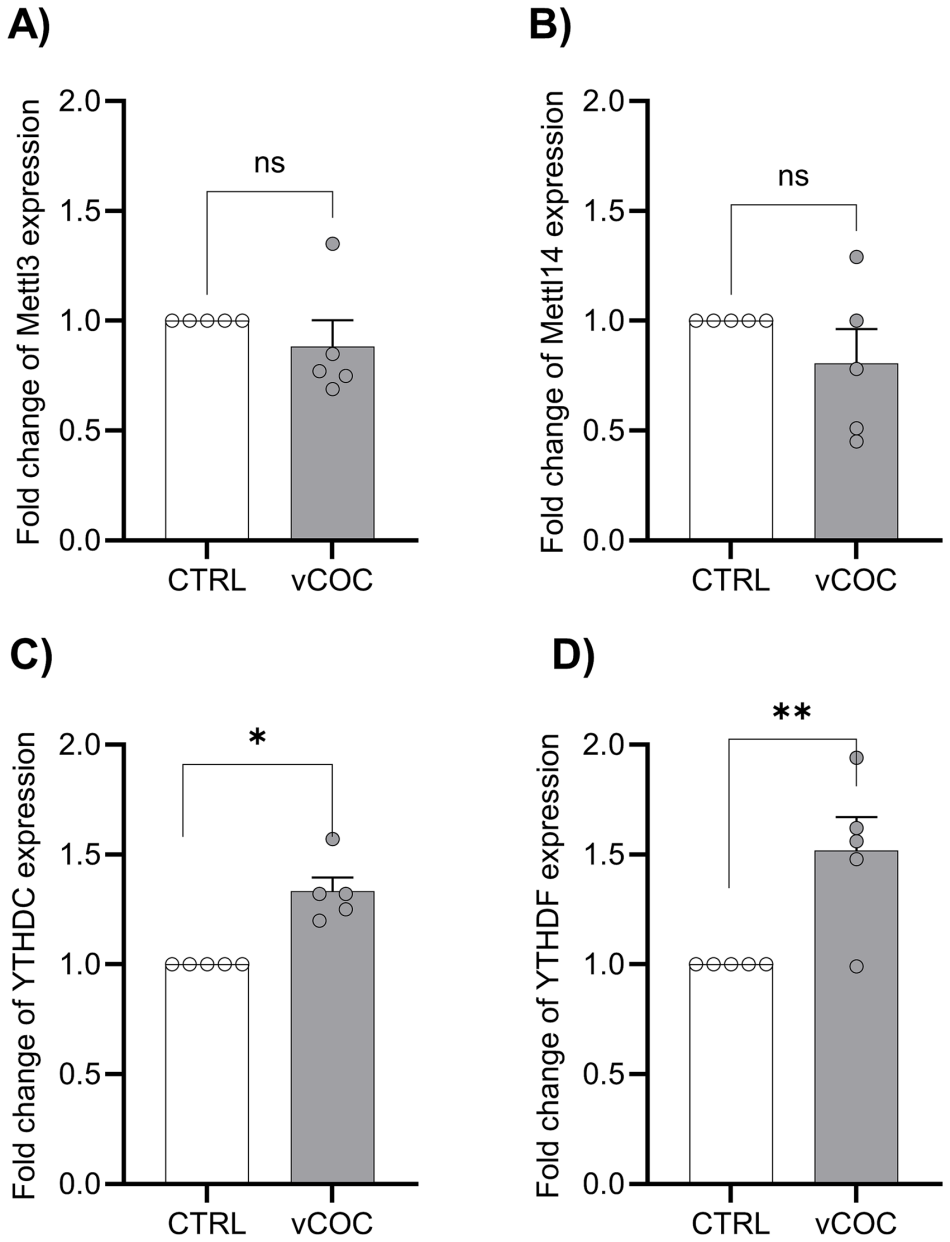
Cocaine administration increased the mRNA levels of nuclear YTHDC and the cytosolic YTHDF reader proteins. Five biological replicates were performed in duplicate for control (CTRL) and experimental (vCOC condition). The CTRL group consisted of 3–5-day-old male Canton-S flies collected from cultivation bottles. The vCOC group consisted of 3–5-day-old male Canton-S flies exposed to two doses of volatilized cocaine (75 μg, 6 h apart) using the FlyBong system. The graphs represent the relative mRNA fold change in the expression of **(A)** Mettl3, **(B)** Mettl14, **(C)** YTHDC, and **(D)** YTHDF. Data are presented as mean ± SEM of fold change 2-ΔΔCt. Statistical analysis was performed using one-way ANOVA followed by Tukey’s post-hoc test calculated using GraphPad Prisma software. * < 0.5, ** < 0.01.

Given the robust increase in m^6^A RNA levels, we next investigated the effects of vCOC on key components of the m^6^A regulatory pathway. Specifically, we quantified mRNA expression levels of the m^6^A methyltransferases Mettl3 and Mettl14, as well as the m^6^A reader proteins YTHDC and YTHDF, in *wt* flies 48 h after two exposures to vCOC using the FlyBong system. vCOC did not alter the transcript levels of the methyltransferases Mettl3 and Mettl14 compared to control flies (Figures 4A,B). In contrast, a significant increase in transcript levels was observed for both reader YTHDC and YTHDF (Figures 4C,D). These findings suggest that vCOC not only affects the levels of m^6^A on RNA, but also selectively enhances the transcript levels of m^6^A readers without altering the core methyltransferase machinery.

### 3.3 Distinct contributions of m^6^A writers and readers to vCOC-induced locomotor sensitization in neurons and glia

Exposure to vCOC induces robust locomotor sensitization (LS) in *D. melanogaster*, a stepwise locomotor activation, which can be quantitatively measured using the FlyBong assay. Given the already described roles of m^6^A in neuronal function and activity-dependent gene regulation, we hypothesized that m^6^A signaling might modulate vCOC-induced behavioral responses. Second, we wanted to determine if there is a selective contribution of neurons or glia to vCOC-induced phenotype of LS. Third, based on the selective vCOC-induced transcript upregulation of the m^6^A readers YTHDC and YTHDF, we investigated their role in vCOC induced phenotype in flies. Using the FlyBong assay, we compared vCOC locomotor responses in flies carrying mutations in Mettl3, YTHDC and YTHDF, as well as cell type specific knockdowns in neurons and glia, to determine if cell specific m^6^A deposition and m^6^A-dependent RNA recognition are required for vCOC-induced locomotor activation.

**Figure 5:**
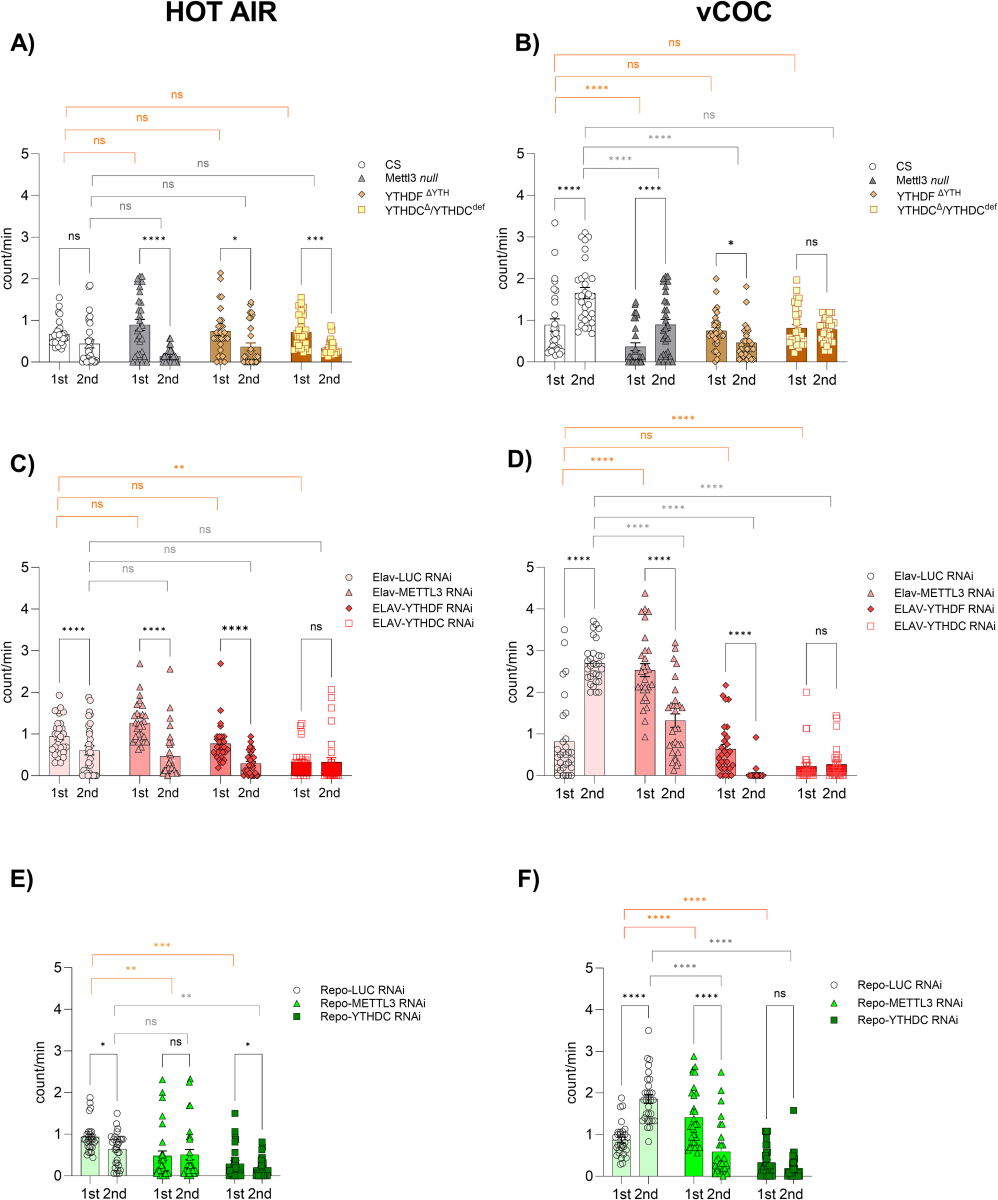
M^6^A deposition and recognition regulate vCOC-induced locomotor sensitization in the FlyBong assay. Locomotor activity (counts/min) was measured in 3–5-day-old male flies during a 30 min period following a 1 min exposure to hot air **(A,C,E)** or volatilized cocaine (vCOC, 75 μg) **(B,D,F)**. locomotor sensitization (LS) is defined as a stepwise increase in activity between 1st (9 a.m.) and 2nd (3 p.m.) exposures. Experimental groups included all cell types mutants: Mettl3, YTHDF, and YTHDC compared to Canton-S (CS) wild type controls (**A,B**; *n* = 16–32 per group, tested in duplicate). Neuronal knockdown: Elav-Gal4 > UAS-Mettl3/YTHDF/YTHDC RNAi vs. UAS-LUC RNAi control (**C,D**; *n* = 16 per group tested in duplicate). Glial knockdown: Repo-Gal4 > UAS-Mettl3/YTHDC RNAi vs. UAS-LUC RNAi control (**E,F**; *n* = 16 per group tested in duplicate). Statistical significance was determined by two-way ANOVA with Tukey’s *post hoc* test. ns = not significant; *, **, ***, **** indicate *p* < 0.05, 0.01, 0.001, 0.0001, respectively. Black denotes within-genotype comparisons between the first (1st) and second (2nd) hot air or vCOC exposures. Orange denotes between-genotype comparisons in response to the first (1st) hot air or vCOC exposure, and gray denotes between-genotype comparisons in response to the second (2nd) hot air or vCOC exposure.

First, we characterized the locomotor behavior in responses to the control condition, a one-minute stream of hot air, in the FlyBong at 9 a.m. (1st) and 3 p.m. (2nd). Locomotor activity was comparable across genotypes and time points, with flies showing a stronger response to the 1st than to the 2nd exposure, opposite to the LS observed following vCOC exposure. No significant differences were detected between control flies and Mettl3, YTHDF, or YTHDC mutants after either the 1st or 2nd hot-air exposure (Figure 5A). Similarly, neuronal knockdown of m^6^A modulators using Elav-GAL4 did not alter locomotor responses to hot air at 1st or 2nd response (Figure 5C). Importantly, glial-specific knockdown of Mettl3 or YTHDC using Repo-GAL4, did significantly affect locomotor activity relative to controls at 1st exposure, and less pronouncedly at 2nd time point (Figure 5E). These results indicate that m^6^A disruption and mock exposure does not induce LS, a hallmark of vCOC exposure.

Two exposures to vCOC revealed that YTHDF and YTHDC mutants failed to exhibit LS, indicating that they are required for the development of LS. In contrast, Mettl3 mutants developed LS, but with lower intensity than *wt* (Figure 5B). This result is in line with the previous molecular analysis (Figure 3) that showed no change in Mettl3 and increase in YTHDF and YTHDC transcript levels after vCOC exposures. Neuronal knockdown of Mettl3, YTHDF, or YTHDC significantly reduced vCOC-induced LS compared to the control (Figure 5D). Glial knockdown of Mettl3 and YTHDC also resulted in attenuated LS (Figure 5F). Taken together these results suggest that Mettl3 plays a complex role in the regulation of locomotor response to vCOC, that is evident only when it is modulated in a tissue-specific manner. In contrast, m^6^A writers showed a more consistent effect, both when mutated and knockdown, in either glial or neuronal cells. Together, these results showed a cell type-specific contributions of m^6^A readers and writers to cocaine-induced locomotor plasticity.

### 3.4 Differential expression analysis showed vCOC-induced suppression of developmental and structural pathways

Given the indication that vCOC exposure alters the level of components of the m^6^A pathway (YTHDC and YTHDF), the levels of m^6^A on polyA-RNA, and that m^6^A perturbation is involved in vCOC-induced LS phenotype, we decided to investigate how vCOC exposure affects gene expression and m^6^A deposition by performing RNA-seq and MeRIP-seq analyses of polyA-enriched RNA from head homogenates, followed by GO and KEGG pathway enrichment (Figure 2).

**Figure 6:**
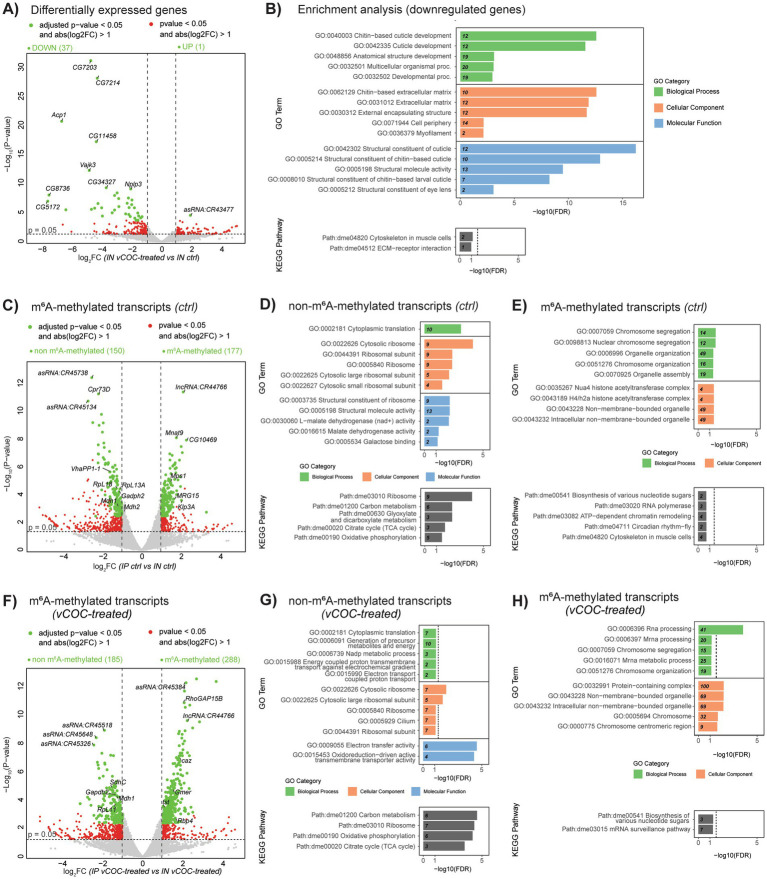
RNA-seq and MeRIP-seq differential gene expression and functional enrichment following vCOC treatment. Volcano plot showing differentially expressed genes between vCOC-treated and control samples: **(A)** Input (IN) CTRL and IN vCOC, **(C)** IN versus immunoprecipitated (IP) in CTRL condition to address m^6^A-enriched and non-enriched in IP relative to IN, and **(F)** IN versus IP in vCOC condition to address m^6^A-enriched and non-enriched in IP relative to IN. The *x*-axis represents log₂ fold change and the *y*-axis represents −log₁₀ adjusted *p*-value. Genes meeting significance thresholds (adjusted *p*-value < 0.05 and |log₂FC| > 1) are highlighted green and red (*p*-value < 0.05 and |log₂FC| > 1). Downregulated genes in differential expression are displayed on the left, unchanged genes in the center, and upregulated genes on the right. **(B)** Gene ontology (GO) and KEGG pathway enrichment analysis of downregulated genes. Bar plots display significantly enriched GO biological process, cellular component, and molecular function terms, as well as KEGG pathways, plotted by −log₁₀(FDR). The dashed line represents the statistical significance cut-off defined as FDR < 0.05. In m^6^A enrichment analysis **(D,G)** are non m6A-enriched genes while **(E,H)** are m^6^A-enriched genes in control **(D,E)** and vCOC **(G,H)** condition.

Transcriptomic analysis of poly(A)-enriched RNA input (IN) revealed widespread gene expression changes following vCOC treatment, with a predominant bias toward downregulation (Figure 6A, Supplementary Table S1). Functional enrichment analysis of downregulated genes, identified processes related to chitin-based cuticle development, extracellular matrix (EMC) organization, and structural components of the cuticle, along with KEGG pathways associated with cytoskeletal organization and ECM-receptor interactions (Figure 6B, Supplementary Table S2). These findings suggest that vCOC exposure could lead to suppression of developmental and structural gene processes.

### 3.5 vCOC exposure preserves metabolic non-m^6^A enrichment while reprogramming m^6^A enrichment toward RNA regulation

To assess m^6^A RNA methylation enrichment, fragmented polyA RNA immunoprecipitated with an anti-m^6^A antibody (IP) was compared with the corresponding input (IN) samples. IP/IN analysis was performed separately for CTRL and vCOC conditions to identify m^6^A-enriched RNA regions, defined as sequences showing significant enrichment in IP relative to IN and representing m^6^A-modified transcripts. Under CTRL conditions, the analysis revealed distinct patterns for m^6^A-enriched and non-enriched transcripts relative to IN (Figure 6C, Supplementary Table S1). Non-enriched-m^6^A transcripts were predominantly associated with housekeeping functions, including ribosomal components, cytoplasmic translation, and core metabolic pathways such as glycolysis and oxidative phosphorylation (Figure 6D, Supplementary Table S2). In contrast, m^6^A-enriched transcripts were associated with regulatory processes, including chromosome segregation, nuclear organization, and macromolecular complex assembly (Figure 6E, Supplementary Table S2), while enriched biological processes encompassed RNA polymerase activity, chromatin remodeling, and circadian rhythm regulation. These data suggest that, under physiological conditions, m^6^A-enriched transcripts are involved in higher-order regulatory and organizational functions rather than core metabolic activities.

Following vCOC exposures, m^6^A-enriched and non-enriched transcripts maintained distinct profiles (Figure 6F, Supplementary Table S1). Non-m^6^A-enriched transcripts, remained similar to CTRL, predominantly associated with metabolic and mitochondrial pathways, including carbon metabolism and oxidative phosphorylation, (Figure 6G, Supplementary Table S2). On the other hand, m^6^A-enriched transcripts exhibited a marked shift toward RNA regulatory functions. GO analysis revealed strong overrepresentation of RNA processing, mRNA metabolic processes, and chromatin-associated functions, while KEGG analysis highlighted pathways related to mRNA surveillance and nucleotide metabolism (Figure 6H, Supplementary Table S2). These findings indicate that vCOC exposure is selectively associated with m^6^A methylation on transcripts involved in RNA regulation and processing, reinforcing a functional separation between m^6^A-dependent regulatory programs and core metabolic transcripts, which remain non-m^6^A-enriched under both conditions.

### 3.6 Differential m^6^A enrichment

Differential m^6^A enrichment was assessed by comparing RNA immunoprecipitated with an anti-m^6^A antibody (IP) between CTRL and vCOC conditions after normalization to the corresponding input (IN) samples. RNA regions showing higher enrichment in vCOC relative to CTRL were classified as hypermethylated, whereas regions with lower enrichment were classified as hypomethylated. The analysis of hypermethylated was performed separately for coding and non-coding RNAs, as these RNA classes fulfill fundamentally distinct biological roles and are subject to different layers of post-transcriptional regulation. In fact, while m^6^A modification of coding transcripts primarily influence mRNA stability, translation, and protein output of the transcripts themselves, m^6^A marks on non-coding RNAs such as lncRNAs, antisense RNAs, and transposable element derived transcripts, are implicated in chromatin regulation, RNA surveillance, and genome stability.

#### 3.6.1 Differential m^6^A enrichment in coding transcripts reflects a shift from neuronal homeostasis to vCOC induced metabolic and redox stress adaptation

**Figure 7:**
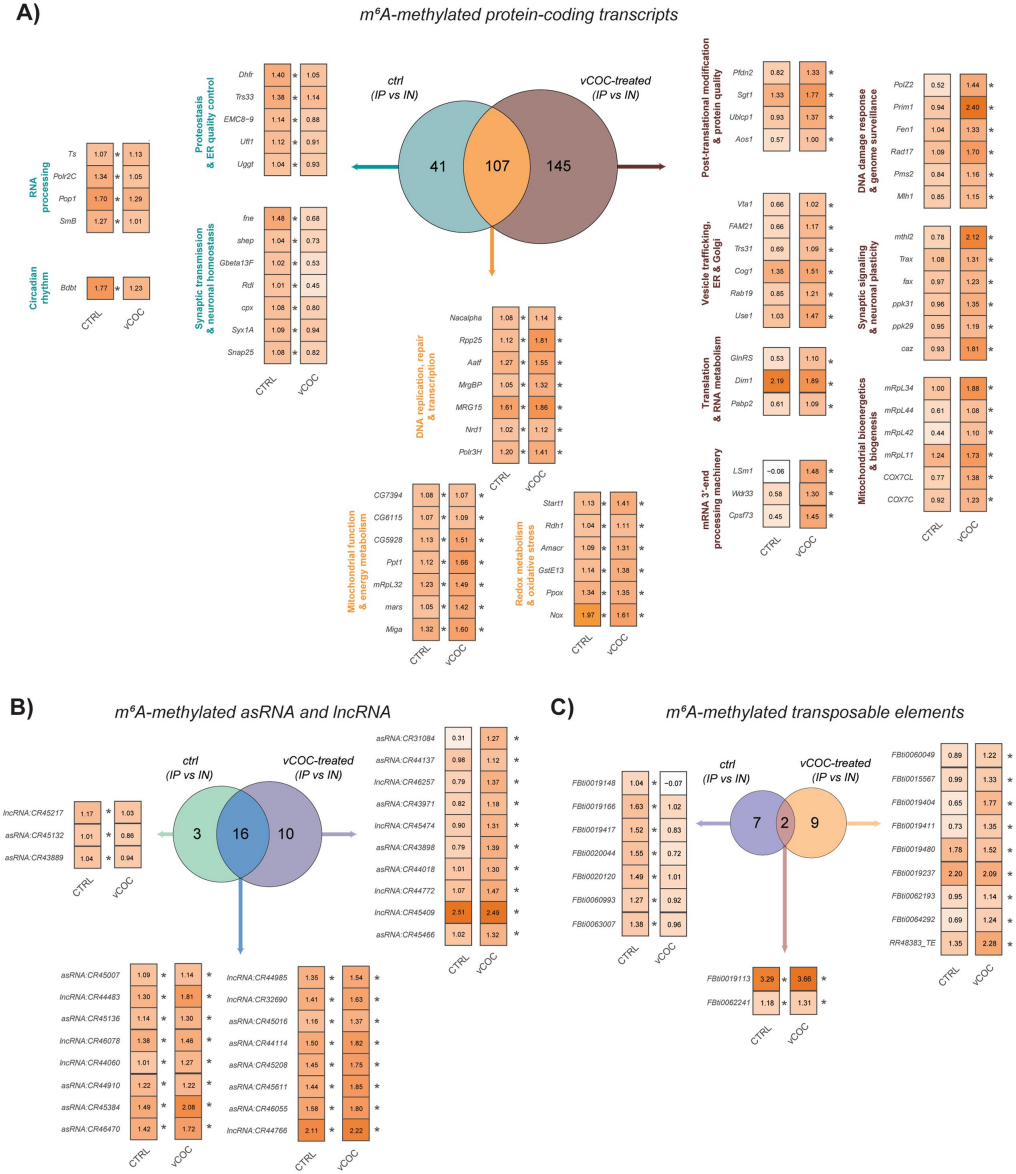
Analysis of differential m^6^A enrichment in CTRL and vCOC conditions displayed separately for coding and non-coding RNAs. Venn diagrams depict the overlap of m^6^A-enriched transcripts (IP vs. IN). Heatmaps display relative m^6^A enrichment (log_2_FC IP/IN) for representative transcripts, with asterisks indicating statistically significant enrichment (*p*adj < 0.05, |log_2_FC| > 1). **(A)** Protein-coding transcripts identified under control (CTRL) and vCOC-treated conditions. A shared core set of transcripts remains methylated in both conditions (orange), while distinct subsets are uniquely enriched in CTRL (green) or following vCOC exposure (brown). Functional categorization of these transcripts reveals that CTRL-specific m^6^A enrichment is associated with circadian regulation, synaptic transmission, neuronal homeostasis, RNA processing, and proteostasis, whereas vCOC-specific m^6^A enrichment shifts toward translation and RNA metabolism, mitochondrial bioenergetics, DNA damage response and genome surveillance, vesicle trafficking, and synaptic plasticity. **(B)** Differential m^6^A enrichment in antisense RNAs (asRNAs) and long non-coding RNAs (lncRNAs) under CTRL and vCOC conditions. A subset of non-coding RNAs shows shared m^6^A enrichment (blue), while additional transcripts are selectively methylated in CTRL (green) and vCOC exposure (violet) conditions, indicating dynamic regulation of regulatory RNA species in response to cocaine. **(C)** Differential m^6^A enrichment of transposable element (TE)–derived transcripts under CTRL and vCOC conditions. A small core set of TEs remains m^6^A-modified in both conditions (brown), whereas the majority display condition-specific enrichment, with increased m^6^A levels following vCOC treatment (orange) compared to CTRL (violet). These data suggest dynamic m^6^A-dependent regulation of TE RNAs, consistent with enhanced RNA surveillance and genome-protective mechanisms during cocaine-induced stress.

Study of the overlapping methylated transcripts between the two conditions, highlighted a core of 107 m^6^A-marked transcripts associated with redox homeostasis, mitochondrial function, and genome integrity (Figure 7A). Presence of genes involved in redox metabolism and oxidative stress (e.g., *Nox, Ppox, GstE13, Amacr, Rdh1, Start1*) suggest that m^6^A constitutively supports cellular defense against oxidative burden, a process particularly relevant in neurons. Similarly, persistent enrichment of mitochondrial and energy metabolism genes (*Miga, mRpL32, Ppt1, CG6115, CG7394*) indicates that m^6^A contributes to maintaining mitochondrial capacity and basal bioenergetics independent of cocaine exposure. Finally, enrichment of transcripts linked to DNA replication, repair, and transcription and protein folding (*Polr3H, Nrd1, MRG15, MrgBP, Aatf, Rpp25, Nacalpha*) suggests a housekeeping role for m^6^A in preserving genome stability and transcriptional fidelity under both basal and challenged states.

Under control conditions, we obtained a list of 41 methylated protein coding transcripts. Some of these are involved in synapse assembly and function and neuronal homeostasis, including *Snap25, Syx1A, cpx, Rdl, shep,* and *fne* (Figure 7A), suggesting a role for m^6^A in stabilizing neuronal development, communication and excitability. Concurrent enrichment of RNA processing and transcriptional machinery (*SmB, Pop1, Polr2C, Ts*), proteostasis/ER quality control pathways (*Uggt, Ufl1, EMC8-9, Trs33, Dhfr*) and circadian function through *Bdbt* further suggests that, under physiological conditions, m^6^A might be involved in fine-tuning neuronal function by coordinating synaptic output with RNA maturation and protein quality control.

vCOC exposure induced a pronounced redistribution of m^6^A by increasing the number of coding m^6^A-enriched transcripts to 145 (Figure 7A). Among these we observed transcripts involved in translation and RNA metabolism, including *Pabp2, LSm1, Dim1,* and *GlnRS*, which indicates an effect of vCOC on post-transcriptional control of protein synthesis, consistent with the rapid cellular adaptations required during cocaine exposure. Notably, vCOC treatment led to a selective enrichment of transcripts of mitochondrial and ribosomal proteins (*COX7C, COX7CL, mRpL11, mRpL42, mRpL44, mRpL34*). This strongly suggests that m^6^A-mediated regulation of mitochondrial translation and respiratory capacity might represent a central adaptive response to cocaine-induced metabolic stress. vCOC-specific m^6^A enrichment also extends to genes involved in synaptic signaling and neuronal development and plasticity (*caz, ppk29, ppk31, fax*), supporting a role for m^6^A in remodeling neuronal circuits underlying behavioral sensitization. At the same time, enrichment of DNA damage response and genome surveillance genes (*Mlh1, Pms2, Rad17, Fen1, Prim1, PolZ2*) suggests that cocaine exposure might induce genotoxic stress, that leads to enhanced post-transcriptional regulation of repair pathways. Additionally, m^6^A targeting of mRNA 3′-end processing machinery (*CPSF73, WDR33, LSm1*), vesicle trafficking, and ER-Golgi pathways (*Use1, Rab19, Cog1, Trs31, FAM21, Vta1*) underscores broad reorganization of RNA handling and intracellular trafficking under vCOC. Finally, enrichment of post-translational modification and protein quality control factors (*Aos1, Ublcp1, Sgt1, Pfdn2*) further supports the idea that m^6^A might coordinates multi-layered adaptive responses, spanning RNA metabolism, translation, mitochondrial function, and proteostasis.

These data suggest a model in which m^6^A maintains neuronal and metabolic homeostasis under basal CTRL conditions, while vCOC exposure triggers a functional reprogramming of m^6^A targets toward translational control, mitochondrial remodeling, and plasticity-related pathways, to enhance translational and mitochondrial adaptation in response to sustained metabolic stress.

#### 3.6.2 vCOC exposure reshapes m^6^A enrichment of non-coding and transposable element RNAs

Beyond protein-coding genes, differential m^6^A enrichment (IP/IN) was performed on antisense RNAs (asRNAs) and long noncoding RNAs (lncRNAs) under CTRL and vCOC-treated conditions (Figure 7B). A core set of 16 noncoding RNAs showed shared m^6^A enrichment in both conditions, indicating a stable, basal m^6^A signature on noncoding transcripts. In contrast, 3 transcripts were uniquely enriched in CTRL and 10 transcripts were uniquely enriched after vCOC. As shown in the heatmaps, the shared core set of lncRNAs showed a generally higher enrichment in the m^6^A fraction in vCOC compared to control (higher IP/IN ratios), This suggests that vCOC exposure not only changes the m^6^A distribution among RNAs but also affects its levels. The larger vCOC-specific set (10 vs. 3) suggests that cocaine selectively promotes m^6^A deposition on additional noncoding RNAs, rather than simply altering the level of methylation of shared targets. This supports a model of stress-induced expansion of the m^6^A regulatory network. Since asRNAs and lncRNAs often regulate transcription, chromatin structure and RNA stability and translation, their increased m^6^A signature under vCOC might likely lead to post-transcriptional fine-tuning of gene regulatory networks, rather than direct changes in steady-state RNA levels.

We also examined m^6^A enrichment (IP/IN) in transposable element (TE) derived transcripts. As shown in Figure 7C only 2 TEs were m^6^A-enriched under both conditions. The majority of m^6^A-enriched TEs were condition-specific: 7 TEs in CTRL and 9 TEs after vCOC exposure. Heatmaps show that vCOC increases m^6^A enrichment magnitude on several TE transcripts (higher IP/IN ratios). TE RNAs are known to be tightly controlled in neurons due to their potential genotoxicity. The shift toward vCOC-specific m^6^A enrichment suggests that cocaine exposure might trigger stress-dependent modification of TE RNAs by the m^6^A machinery. m^6^A deposition on TE RNAs is thought to promote: nuclear retention, RNA decay and controlled translation. Thus, increased or redistributed m^6^A on TEs upon vCOC exposure likely serves as a protective buffering mechanism, preventing excessive TE mobilization or aberrant RNA accumulation during cocaine-induced stress.

vCOC selectively expands and amplifies m^6^A enrichment on noncoding and transposable element-derived RNAs, rather than globally altering their methylation, indicating active remodeling of the noncoding m^6^A landscape under cocaine exposure. This cell stress-dependent redistribution likely enhances post-transcriptional control of regulatory and potentially genotoxic RNAs, positioning m^6^A as a buffering mechanism that stabilizes gene regulatory networks and genome integrity during cocaine-induced neuronal stress.

## 4 Discussion

We identified m^6^A RNA methylation as post-transcriptional mechanism potentially engaged by volatilized cocaine (vCOC) exposure to shape neuronal adaptation and behavior in *Drosophila,* through differential m^6^A enrichment of both coding and non-coding RNAs. Our study demonstrates that vCOC increases global m^6^A RNA levels in *Drosophila* head homogenates, particularly on poly(A)-enriched transcripts, without altering transcript levels of the core methyltransferases Mettl3 and Mettl14, consistent with observations in mammals (Xue et al., 2021; Xu et al., 2020). These observations indicate that the accumulation of m^6^A is unlikely due to an increase in writers abundance, but that it rather reflects changes in targeting, stabilization, or downstream processing of methylated RNAs. In *Drosophila*, where canonical m^6^A demethylases such as ALKBH5 and FTO have not been functionally validated, m^6^A dynamics are thought to rely primarily on regulated deposition and reader-mediated interpretation rather than on active demethylation (Dai and Asgari, 2023). Although the existence of a context-dependent or as-yet unidentified demethylase pathway cannot be excluded, our data suggest that vCOC predominantly engages the m^6^A system by reshaping how methylation marks are interpreted rather than removed. Consistent with this model, vCOC exposure is accompanied by increased transcript levels of m^6^A reader proteins YTHDC and YTHDF, which would modulate the cell’s capacity to decode the methylation landscape. YTH family proteins are established effectors of m^6^A-dependent regulation, controlling RNA splicing, export, stability, and translation (Roundtree et al., 2017; Chen et al., 2023). Enhanced reader availability may therefore potentiate downstream RNA regulatory processes relevant to neuronal function and plasticity, consistent with reader-associated adaptations reported in mammalian models of cocaine exposure (Xue et al., 2021). Functionally, m^6^A recognition rather than global m^6^A deposition, is essential for vCOC-induced locomotor sensitization (LS), as loss of either YTHDC or YTHDF abolishes vCOC induced behavioral response.

At the whole-animal level, mutation of the methyltransferase Mettl3 did not abolish vCOC-induced LS. This suggests that reducing global m^6^A deposition alone is not sufficient to disrupt behavioral plasticity. Instead, these results imply that m^6^A-dependent effects on behavior likely depend on selective, reader-mediated interpretation of specific transcripts rather than on the overall abundance of m^6^A marks. This outcome is consistent with the lack of vCOC-induced changes in Mettl3 transcript levels. However, cell type-specific manipulations revealed a more nuanced role for m^6^A deposition: Neuronal depletion of Mettl3 produced strong attenuation of LS, while glial knockdown also reduced the response, suggesting that coordinated m^6^A regulation across neurons and glia contributes to behavioral output. In this framework, loss of Mettl3 in a single cell type produces only partial effects, whereas systemic disruption alters the balance of m^6^A-dependent programs across cell populations, consistent with prior observations in aging *Drosophila* (Perlegos et al., 2024).

Because the primary behavioral experiments employed whole body genetic m^6^A pathway mutants, RNA-seq was performed on whole-head RNA to capture global epitranscriptomic changes. In fact, the cell type-specific manipulations were included as complementary approaches to assess relative sensitivity to m^6^A disruption, rather than to assign these changes to specific cellular populations only at behavioral level. Tissue collection was performed 48 h after vCOC exposure to capture late, sustained transcriptional and epitranscriptomic state-changes associated with behavioral adaptation, rather than acute cocaine-induced responses. RNA-seq analysis of poly(A)-enriched RNA comparing CTRL and vCOC-treated flies revealed a limited transcriptional response, with only 37 genes downregulated and a single gene upregulated following vCOC exposure. This relatively mild effect likely reflects the timing of tissue collection, which occurred 48 h after vCOC administration, a stage at which acute transcriptional responses are expected to have subsided. Functional enrichment analysis of the differentially expressed genes, showed predominant downregulation of pathways related to chitin biosynthesis and degradation, along with KEGG enrichment for extracellular matrix (ECM)-receptor interaction pathways, which govern cell-ECM communication, adhesion, and structural integrity. In contrast to previous whole-brain and single-cell studies, that examined immediate responses to cocaine and reported rapid and bidirectional changes in neuronal gene expression (Baker et al., 2021a, 2021b), our delayed time point captures a distinct transcriptional state characterized by selective repression of cellular architecture and structural integrity. This pattern suggests that early cocaine-induced transcriptional activation resolves over time, leaving a longer-lasting suppression of developmental and structural genes rather than sustained changes in neuronal transcription.

m^6^A-enrichment analysis under CTRL conditions, showed that the m^6^A marks are enriched on a subset of transcripts that include genes involved in circadian regulation, synaptic homeostasis, RNA processing, proteostasis, and nuclear organization. In contrast, housekeeping and metabolic transcripts (e.g., glycolysis, oxidative phosphorylation) are largely non-m^6^A-enrich under CTRL conditions. Therefore, we could conclude that the m^6^A landscape supports neuronal signaling, circadian rhythm, and RNA regulatory networks.

vCOC exposure is associated with a pronounced reprogramming of the m^6^A landscape, shifting the m^6^A signature from transcripts supporting basal neuronal stability toward RNAs involved in RNA processing, translation, mitochondrial bioenergetics, genome surveillance, and synaptic plasticity, while non-m^6^A transcripts continue to mark core metabolic and maintenance functions. This adaptive reprogramming is accompanied by elevated m^6^A levels in poly(A)-enriched RNA without corresponding changes in Mettl3 transcript abundance (Figure 3), suggesting that vCOC might possibly modulate Mettl3 activity, substrate targeting or substrate stability. In parallel, reader engagement shifts in a manner consistent with this redistribution: upregulation of the cytoplasmic reader transcript levels YTHDF (Figure 3) might enhance the stabilization and translation of stress-adaptive mRNAs, particularly those involved in mitochondrial and translational remodeling. On the other hand, increased transcript level of the nuclear reader YTHDC (Figure 3) may support RNA processing, decay, or export of newly targeted transcripts. This indicates that vCOC could be associated with a selective relocation of m^6^A marks toward pathways that promote cellular adaptation, enhancing mitochondrial capacity, translational control, RNA quality surveillance, and neuronal plasticity, rather than inducing a uniform alteration in RNA methylation. Our findings that vCOC reshapes the m^6^A landscape in the *Drosophila* brain, align with emerging evidence that m^6^A serves as a dynamic, stress-responsive regulator of neuronal function. In the adult fly brain, Mettl3-dependent m^6^A marks and the nuclear reader YTHDC1 were shown to fine-tune stress-responsive transcripts during heat shock, with reduced methylation or reader activity enhancing stress resilience (Perlegos et al., 2022). Similarly, in aging and disease contexts, m^6^A levels increase in a cell type-specific manner, regulating transcripts associated with neuronal signaling, RNA processing, and synaptic homeostasis, and influencing organismal outcomes such as survival under chronic stress (Perlegos et al., 2024). Consistent with these studies, vCOC exposure selectively enriches m^6^A on coding RNAs involved in RNA processing, mitochondrial bioenergetics, genome surveillance, and synaptic plasticity, without broadly altering housekeeping or metabolic transcripts. Importantly, vCOC-induced behavioral plasticity depends on m^6^A readers YTHDC and YTHDF, rather than core methyltransferases, highlighting that reader-mediated interpretation of m^6^A marks possibly drives functional adaptation. These data indicate a conserved principle: m^6^A dynamically modulates brain transcriptomes in response to diverse stressors, directing adaptive molecular programs and shaping organismal outcomes without globally reshaping metabolism.

Analysis of the common m^6^A landscape in both CTRL and vCOC conditions, identified transcripts connected to redox and mitochondrial activity, rather than oxidative damage. Core m^6^A-modified genes include primary redox enzymes and reactive oxygen spaces (ROS) generators such as Nox, GstE13, Ppox, Rdh1, Amacr, and Start1, which together define cellular redox tone and directly influence mitochondrial respiration and metabolic flux. In parallel, m^6^A enrichment was observed on genes involved in mitochondrial dynamics and translational adaptation, including Miga, mars, and mRpL32, suggesting redox-sensitive remodeling of mitochondrial morphology and protein synthesis capacity. Importantly, these mitochondrial signals converge on nuclear and stress-responsive pathways through factors such as Aatf, MRG15, and MrgBP, consistent with activation of mitochondrial-to-nuclear retrograde signaling (Jiang et al., 2024; Devoucoux et al., 2022; Cardamone et al., 2018). Collectively, this gene network supports a model in which vCOC affects the mito-redox signaling loop, characterized by balanced ROS production, mitochondrial adaptation, and transcriptional reprogramming, rather than inducing oxidative stress. Such ROS regulation is commonly associated with neuronal stress adaptation and plasticity, supporting the idea that m^6^A marks selectively tune redox-mitochondrial communication during cocaine-induced behavioral adaptation. However, these enrichment patterns should be interpreted with caution, as they are hypothesis-generating and indicate potential pathway involvement rather than direct evidence of functional mechanisms.

The selective enrichment of m^6^A on DNA repair related transcripts in vCOC samples in contrast to CTRL is probably more consistent with a secondary, adaptive response to chronic cellular stress rather than a primary genotoxic or apoptotic effect. Cocaine exposure is well known to elevate monoamine signaling, particularly dopamine, which drives increased neuronal activity and contributes to redox and metabolic stress (Filošević Vujnović et al., 2023a, 2023b). As shown in the supplementary dataset, vCOC-specific m6A enrichment occurs on stress-responsive translational regulators (eIF2α, eIF2Bβ), mitochondrial respiratory and translational components (COX7C, COX7CL, mtTFB2, Ant2, mRpL11/42/44), and RNA turnover machinery (Cpsf73, Wdr33, LSm1). Within this stress context, the emergence of m^6^A enrichment on genome maintenance factors, including checkpoint signaling (Rad17), mismatch repair (Mlh1, Pms2), and replication stress associated enzymes (Fen1, Prim1, PolZ2), suggests coordination of genome surveillance pathways in response to sustained oxidative, metabolic, and activity-driven stress rather than acute DNA damage. Importantly, the CTRL m^6^A enriched profile lacks any enrichment of DNA repair or checkpoint genes and is instead composed by transcripts involved in synaptic homeostasis, RNA processing, and proteostasis, arguing against constitutive m^6^A regulation of genome maintenance. Circadian genes, which govern the body’s internal clock and orchestrate daily rhythms in physiology and behavior, also influence key cellular processes such as mitochondrial metabolism, DNA repair, and protein translation (Yang et al., 2025; Cela et al., 2025; Kim and Sun, 2024). Interestingly, these transcripts are not m^6^A-enriched following vCOC exposure, suggesting that circadian genes may coordinate adaptive cellular responses and potentially interact with m^6^A RNA methylation to fine-tune neuronal function during periods of heightened neuronal activity induced by vCOC (Chauhan et al., 2026; Roignant and Soller, 2017; Lokody, 2014; Hastings, 2013). Moreover, the absence of apoptotic signatures in CTRL and the preferential targeting of replication and repair fidelity factors are more consistent with low-level, chronic cellular stress adaptation than cell death pathways. These findings support a model in which cocaine-induced monoamine signaling elevates neuronal activity and redox/metabolic stress, triggering a redistribution of m^6^A toward transcripts that stabilize translation, mitochondrial function, RNA metabolism, and DNA repair while potentially being coordinated with circadian timing to maintain cellular viability under prolonged challenge.

MeRIP-seq analysis further revealed that under basal conditions, transcripts encoding key circadian regulators, including Bride of Doubletime (BDBT) and PAR domain protein 1 (PDP1), are enriched for m^6^A. Mettl3-dependent methylation of these mRNAs supports circadian homeostasis by regulating period (PER) protein stability and clock/cycle (CLK/CYC)-driven transcription. Following vCOC exposure, although Mettl3 transcript levels remain unchanged, m^6^A enrichment is selectively reduced on these circadian transcripts and redistributed toward RNAs associated with stress responses, mitochondrial function, and translational control. This shift suggests a dynamic post-transcriptional reallocation that prioritizes adaptive metabolic and stress-related programs at the expense of circadian precision. Consistent with this model, disruption of circadian function, either through neuron- or glia-specific Mettl3 depletion or mutation of the core clock gene *period* (Andretic et al., 1999), abolishes LS, indicating that intact, system-wide m^6^A-dependent regulation of circadian transcripts is required for cocaine-induced behavioral plasticity.

m^6^A marks might be interpreted by the nuclear reader YTHDC to regulate splicing and RNA processing of circadian transcripts, and by the cytoplasmic reader YTHDF to control the stability and translation of stress- and metabolism-related RNAs. Consistent with this division of labour, global loss of either YTHDC or YTHDF abolishes vCOC-induced LS, a phenotype that is recapitulated by neuron- and glia-specific knockdown. Notably, vCOC exposure leads to increased YTHDC and YTHDF transcript levels, as detected by RT-qPCR, suggesting that reader upregulation may represent a compensatory response to the expanded and redistributed m^6^A landscape. This suggests that both loss of reader function and dysregulated reader availability are sufficient to disrupt the post-transcriptional programs required for adaptive behavioral plasticity. While vCOC exposure correlated with altered m^6^A profiles and disruption of m^6^A components impaired LS, our data do not directly establish that cocaine-induced m^6^A remodeling causally drives the behavioral phenotype. Future studies will be required to determine whether these epitranscriptomic changes are mechanistically responsible for the observed behavioral effects. Accordingly, our findings support a role for YTH-domain proteins in the behavioral response to vCOC, while the specific RNA targets and underlying mechanisms remain to be determined.

The coordinated m^6^A remodeling observed in vCOC samples extends beyond protein-coding transcripts to encompass noncoding RNAs and transposable element (TE) RNAs, indicating a broader epitranscriptomic adaptation to cocaine-induced stress. Differential enrichment analyses revealed that while a core set of 16 ncRNAs is m^6^A-modified under conditions, cocaine exposure increases the number of methylated lncRNAs and antisense RNAs (10 vCOC-specific vs. 3 control-specific), with elevated IP/IN ratios suggesting stronger methylation. These noncoding transcripts modulate transcription, chromatin structure, RNA stability, and translation, suggesting that m^6^A may fine-tune gene regulatory networks in response to cocaine without altering steady-state RNA levels (Zhang and Wang, 2023; Xue et al., 2021; He and Lan, 2021; Xu et al., 2020). This remodeling parallels the vCOC-specific enrichment in DNA repair, translational, and mitochondrial genes, consistent with in the hypothesis that cocaine-induced monoamine signaling elevates neuronal activity and redox/metabolic stress, propagating to genome stress and triggering m^6^A retargeting. Similarly, TEs exhibit dynamic m^6^A regulation: while a small core of TE transcripts remains methylated in both conditions, the majority show condition-specific enrichment, with 9 TEs uniquely m^6^A-modified under vCOC.

Together these results suggenst the potentian involvement of the Mettl3-m^6^A-YTHDC/YTHDF axis in circadian and stress-responsive gene expression modulation to support vCOC-induced LS. In line with this, cocaine has been shown to increase m^6^A modification on RNAs involved in synaptic plasticity and dopamine signaling in mammalian models (Xue et al., 2021), and disruption of YTHDC or YTHDF is predicted to impair RNA splicing, stability, and translation within reward-related neural circuits. Such defects likely underlie the reduced vCOC-induced LS observed here, as well as the learning and memory impairments reported in other *Drosophila* m^6^A reader studies (Kan et al., 2021).

## Data Availability

The datasets presented in this study can be found in online repositories. The names of the repository/repositories and accession number(s) can be found in the article/Supplementary material.

## Footnotes

1
https://bioinfogp.cnb.csic.es/tools/venny/
